# Calculated parenteral initial therapy of bacterial infections: Bacterial meningitis

**DOI:** 10.3205/id000051

**Published:** 2020-03-26

**Authors:** Pramod M. Shah, Reinhard Brodt, Thomas A. Wichelhaus, Roland Nau

**Affiliations:** 1 Frankfurt am Main, Germany; 2 Med. Klinik II / Infektiologie, Universitätsklinikum Frankfurt am Main, Germany; 3 Institut für Medizinische Mikrobiologie und Krankenhaushygiene, Universitätsklinikum Frankfurt, Deutschland; 4 Geriatrisches Zentrum, Evangelisches Krankenhaus Göttingen-Weende, Göttingen, Germany

## Abstract

This is the thirteenth chapter of the guideline “Calculated initial parenteral treatment of bacterial infections in adults – update 2018” in the 2^nd^ updated version. The German guideline by the Paul-Ehrlich-Gesellschaft für Chemotherapie e.V. (PEG) has been translated to address an international audience.

Bacterial meningitis is a life-threatening infectious disease with high mortality and disability rates requiring prompt initiation of antimicrobial treatment to lower these rates.

## Zusammenfassung

Dies ist das 13. Kapitel der von der Paul-Ehrlich-Gesellschaft für Chemotherapie e.V. (PEG) herausgegebenen S2k Leitlinie „Kalkulierte parenterale Initialtherapie bakterieller Erkrankungen bei Erwachsenen – Update 2018“ in der 2. aktualisierten Fassung.

Die bakterielle Meningitis ist eine lebensbedrohliche Infektionskrankheit mit hohen Mortalitäts- und Invaliditätsraten, die den sofortigen Beginn einer antimikrobiellen Behandlung erfordert, um diese Raten zu senken.

## Introduction

Acute bacterial meningitis is characterized by the clinical cardinal symptoms of fever, headaches and irritation of the meninges (meningism). In addition, confusion, epilepsies or coma may also feature in the clinical presentation [1], [2]. Acute bacterial meningitis must be distinguished from viral meningitis. The most common causes of acute bacterial meningitis acquired outside of hospitals are meningococci and pneumococci. Less common are *Haemophilus influenzae*, Listeria and *Mycobacterium tuberculosis*. According to the Infectious Disease Yearbook of Notifiable Diseases 2014, 275 cases of meningococcal meningitis were reported in Germany (incidence 0.3 per 100,000) [3]. Of these, 71.3% belonged to serogroup B, 17.1% to serogroup C, 7.5% to serogroup Y and 4.2% to serogroup W. Data from Germany on the incidence of meningitis caused by pneumococci or listeria are not available. Austria reported an incidence of 0.42/100,000 for pneumococci in 2013 and an incidence of 0.71/100,000 for meningococci (http://www.ages.at/). The pathogen spectrum is age-dependent. Under certain circumstances (post-operative, CSF-associated and immunosuppressed patients), other pathogens such as staphylococci, enterobacteria and pseudomonads can also cause bacterial meningitis.

Meningitis associated with craniofacial infections is mainly caused by pneumococci and other streptococci. There may also be organic manifestations in the CNS in the context of other septic infectious diseases, especially in leptospirosis or *Borrelia burgdorferi* infections. Subacute or chronic meningitic syndrome is caused in particular by mycobacteria, *Candida* species, *Cryptococcus neoformans*, *Coccidioides immitis* and *Treponema pallidum*. An atypical progression of meningitis can be expected under severe immunosuppression.

## Diagnostics

For all patients blood cultures should be taken. Depending on the location of concomitant infections, it may be necessary to obtain a throat swab, bronchial secretions, urine or a wound swab. 

A diagnosis of bacterial meningitis can be proven through lumbar puncture and examination of the cerebrospinal fluid. Granulocytic pleocytosis in excess of 1,000 cells/µl, CSF protein above 100 mg/dl, CSF lactate greater than 3.5 mmol/l and a CSF/serum glucose ratio below 0.3 is typical. Methylene blue staining and Gram staining of the CSF sediment may provide general clues (Gram-negative rods or cocci, Gram-positive rods or cocci). For additional diagnostics, antigen detection methods from the CSF and urine, PCR from the CSF (especially in cases of suspected tuberculous meningitis or for the detection/exclusion of viral CNS infections), CRP/PCT measurement in serum and differential blood counts have proven useful [4]. In subacute meningitis and encephalitis, in particular in neuroborreliosis, the detection of pathogen-specific antibody synthesis in the CNS through determining the CSF/serum antibody index is of great importance [5].

Patients with severe disturbed consciousness, patients with a focal neurological deficit (for example hemiparesis), patients with epileptic seizures within the last few days or immunosuppressed patients who are strongly suspected of having bacterial meningitis should be referred to initial treatment with parenteral antibiotics and dexamethasone immediately after taking the blood sample (for blood culture). Subsequently, a cranial CT and, if the CT findings do not indicate otherwise, a lumbar puncture will be performed [6]. A Swedish study indicates that even in patients with disturbed consciousness CSF can be taken without prior cerebral imaging to determine the etiology. Reducing the time gap between admittance and first dose of antibiotics this way seems to more than outweigh the risk of herniation due to CSF removal [7].

## Treatment

Delayed initiation of antibiotic treatment is associated with an unfavorable prognosis [8], [9]. Based on the pathogen spectrum of community-acquired bacterial meningitis, calculated initial treatment (Table 1 (Tab. 1)) with a Group 3a cephalosporin in combination with ampicillin (effect on Listeria) should be initiated [10]. For nosocomial bacterial meningitis and infected CSF drainage, initial treatment consists of vancomycin plus meropenem or vancomycin plus ceftazidime. If a pathogen can be detected, further treatment will be based on the results of the microbiological testing (Table 2 (Tab. 2)). Infected CSF drainage must usually be removed and replaced with external CSF drainage. The duration of treatment with unknown pathogens and meningitis caused by *Haemophilus influenzae* or *Streptococcus pneumoniae* should not be less than 10 days and in meningococcal meningitis not less than 7 days. In patients with meningitis caused by Listeria, *Staphylococcus aureus*, *Pseudomonas aeruginosa* or enterobacteria, antibiotic treatment lasts 3 weeks.

Fever or an increase in pleocytosis in sterile CSF alone is no reason for prolonging treatment. A final puncture on or after the end of the treatment is not required in cases of uncomplicated progression.

In pathogens with reduced sensitivity to antibiotics, intraventricular antibiotic treatment may be necessary to eliminate the CNS pathogens. At present, no drug is approved for intraventricular administration in Germany and randomized trials which could lead to an improvement of the treatment outcome through intraventricular treatment do not exist. Intraventricular administration of antibiotics thus constitutes an attempted cure. Antibiotics for which intraventricular use makes sense due to low crossover into the CSF even in cases of highly inflammatory meninges and high systemic toxicity and for which experience reports have been published are listed in Table 3 (Tab. 3) [11]. Managing intrathecal treatment by measuring CSF concentrations would appear to be sensible [12].

In a Cochrane analysis of 25 studies on the use of corticosteroids in bacterial meningitis, there was a significant reduction in mortality in countries with high medical standards (diagnosis and treatment) when pneumococci were the causative pathogens (RR 0.84, 95% CI 0,72– 0,98) but not in *Haemophilus influenzae* or *Neisseria meningitidis* meningitis [13]. Treatment with corticosteroids also resulted in a significant reduction of damage to hearing (RR 0.74, 95% CI 0.63–0.8) and neurological consequential damage (RR 0.83, 95% CI 0.69–1.00). 

Dexamethasone 10 mg i.v. 4 times a day over 4 days plus empirical antibiotic administration is the recommended initial treatment regimen for adult patients suspected of having bacterial meningitis.

Insufficient data are available for patients with nosocomial meningitis and immunosuppressed patients with bacterial meningitis, so adjuvant dexamethasone administration is not recommended. For further adjuvant strategies which have been proven effective in animal experiments, there is insufficient experience in human adults [14], [15]. Due to published negative studies, routine adjuvant therapy with paracetamol, glycerol or hypothermia in bacterial meningitis is not recommended [16], [17], [18].

In tuberculous meningitis, adjuvant administration of dexamethasone or prednisolone improves outcomes [19]. According to a common formula, adults and adolescents in stages II and III receive dexamethasone intravenously 0.4 mg/kg/day in week 1, 0.3 mg/kg/day in week 2, 0.2 mg/kg/day in week 3 and 0.1 mg/kg/day in week 4, followed by oral dexamethasone for 4 weeks with a daily dose reduction of 1 mg per week. In stage I, intravenous administration of dexamethasone 0.3 mg/kg/day in week 1, 0.2 mg/kg/day in week 2, followed by dexamethasone 0.1 mg/kg/day orally in week 3, 3 mg/day orally in week 4, 2 mg/day orally in week 5 and 1 mg/day in week 6 [20]. Alternatively, a prednisolone regimen may be considered, starting at 60–80 mg/day decreasing for 4–6 weeks. Dosage recommendations for tuberculous meningitis see Table 4 (Tab. 4).

For thrombosis prophylaxis, low-dose heparinization and for gastric protection, the application of proton pump inhibitors is recommended.

## Prophylaxis

The most common cause of meningitis after splenectomy is *Streptococcus pneumoniae*, followed by other encapsulated bacterial species. It is therefore recommended that active vaccination using a pneumococcal, hib- and meningococcal vaccine is carried out prior to removal of the spleen (in emergencies also after surgery). Concerning the further indications for *Haemophilus*-, pneumococcal and meningococcal vaccination, refer to the homepage of the Standing Vaccination Commission of the Robert Koch-Institute (http://www.rki.de/nn_199596/DE/Content/Infekt/Impfen/impfen.html).

Based on current resistance data, people in close contact with patients with meningococcal meningitis acquired in Germany receive antimicrobial prophylaxis up to 10 days after the last patient contact using ciprofloxacin, rifampicin or ceftriaxone [21]. Adults (except pregnant women) receive ciprofloxacin (single dose 500–750 mg p.o.) or alternatively rifampicin (600 mg every 12 hours for 2 days). Pregnant women receive ceftriaxone (single dose 250 mg, i.m.). Children receive rifampicin (10 mg/kg bodyweight every 12 hours for 2 days, p.o.). There have been reports of rifampicin (including as prophylaxis) leading to rapid development of resistance [22]. An increase in ciprofloxacin resistance is to be expected in Neisseria meningitidis strains from southern and western Europe as well as from southeast Asia [23], [24], [25].

People in close contact with patients with Haemophilus influenzae meningitis receive antimicrobial prophylaxis up to 10 days after the last patient contact [26]: Adults (except pregnant women) receive rifampicin (600 mg every 24 hours for 4 days, p.o.); children under the age of 12 receive rifampicin (20 mg/kg body weight every 24 hours for 4 days, p.o.).

## Note

This is the thirteenth chapter of the guideline “Calculated initial parenteral treatment of bacterial infections in adults – update 2018” in the 2^nd^ updated version. The German guideline by the Paul-Ehrlich-Gesellschaft für Chemotherapie e.V. (PEG) has been translated to address an international audience.

## Competing interests

The authors declare that they have no competing interests.

## Figures and Tables

**Table 1:**
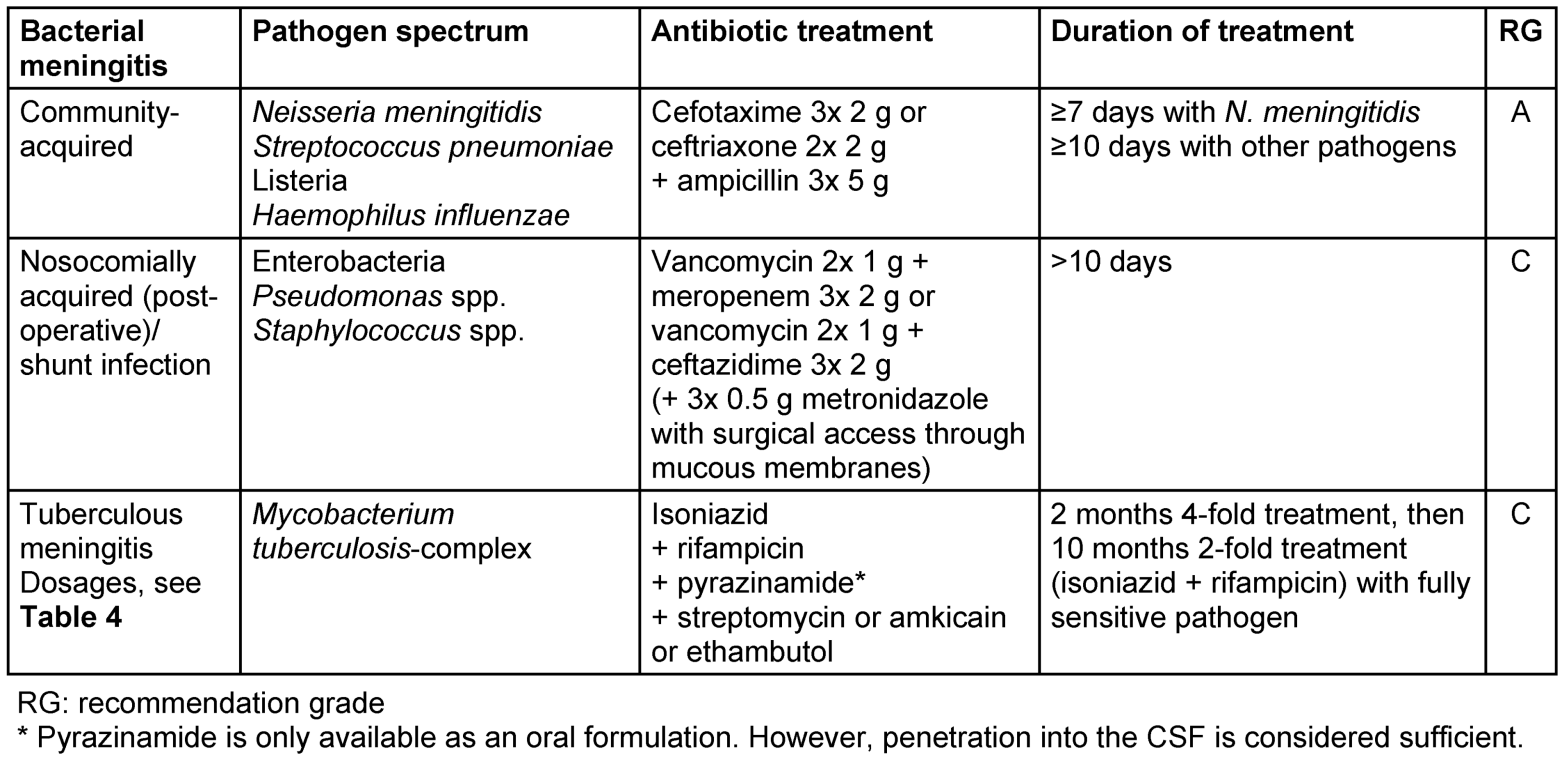
Calculated antibiotic treatment of bacterial meningitis in adults. The recommendations of the S2k guideline on the treatment of tuberculosis by the German Central Committee for the Control of Tuberculosis e.V. on behalf of the German Society of Pneumology and Respiratory Medicine e.V.

**Table 2:**
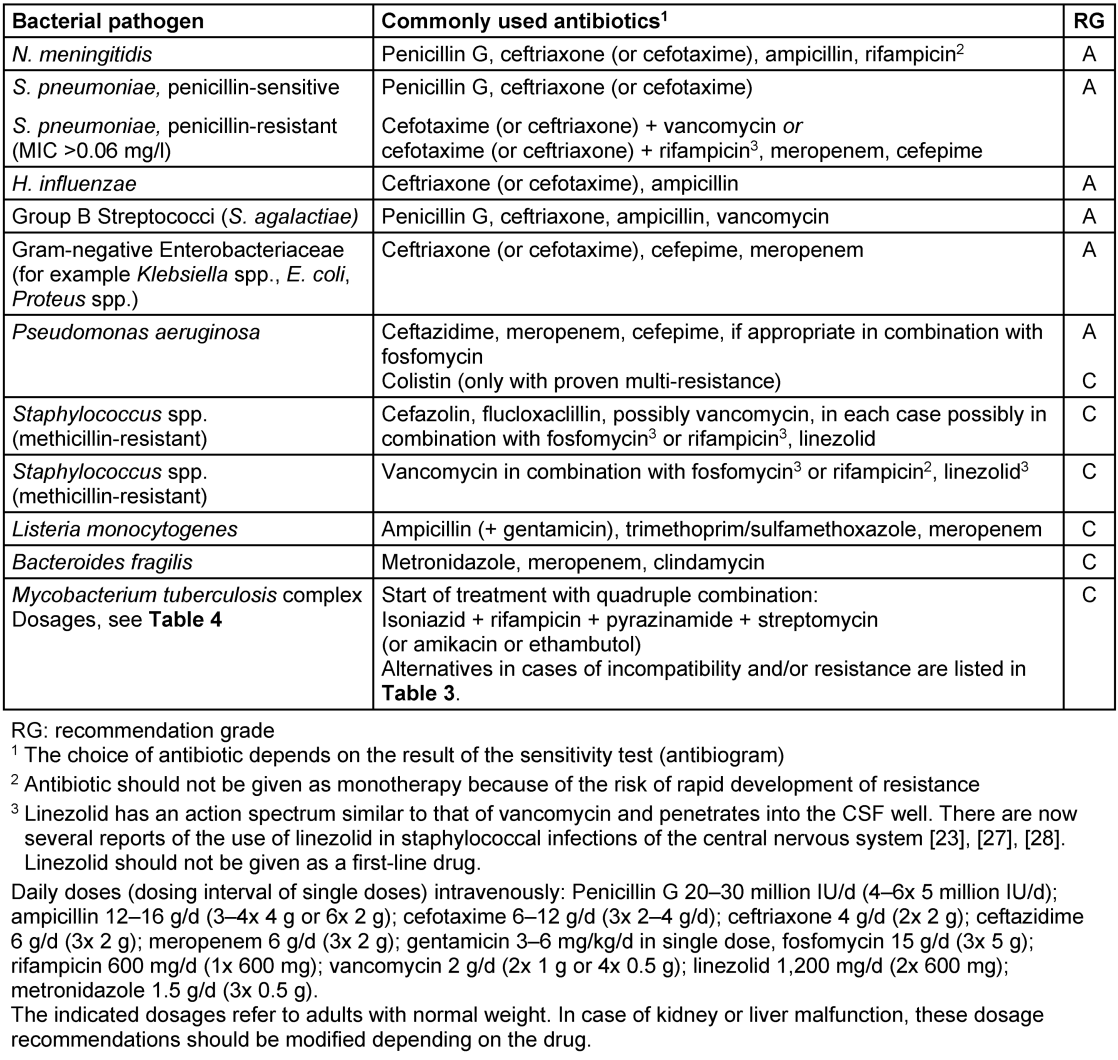
Targeted antibiotic treatment of bacterial meningitis in adults [according to guidelines of the German Society of Neurology (http://www.dgn.org)]

**Table 3:**
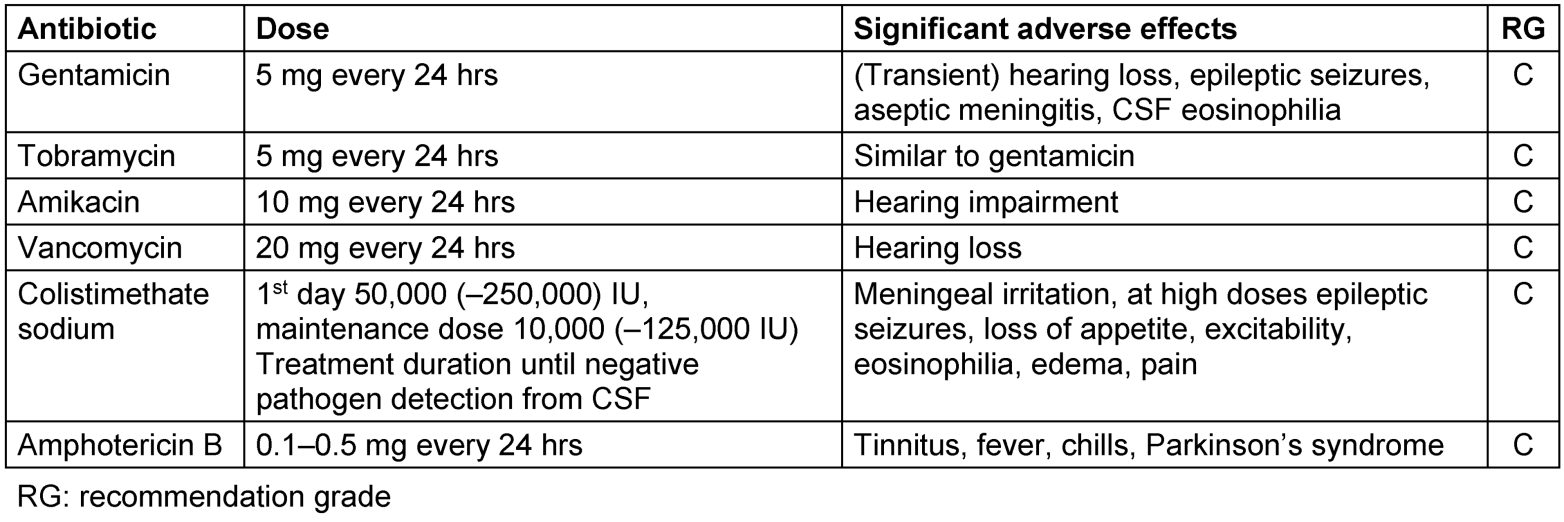
Intraventricular antibiotic treatment (usually simultaneous systemic treatment is required)

**Table 4:**
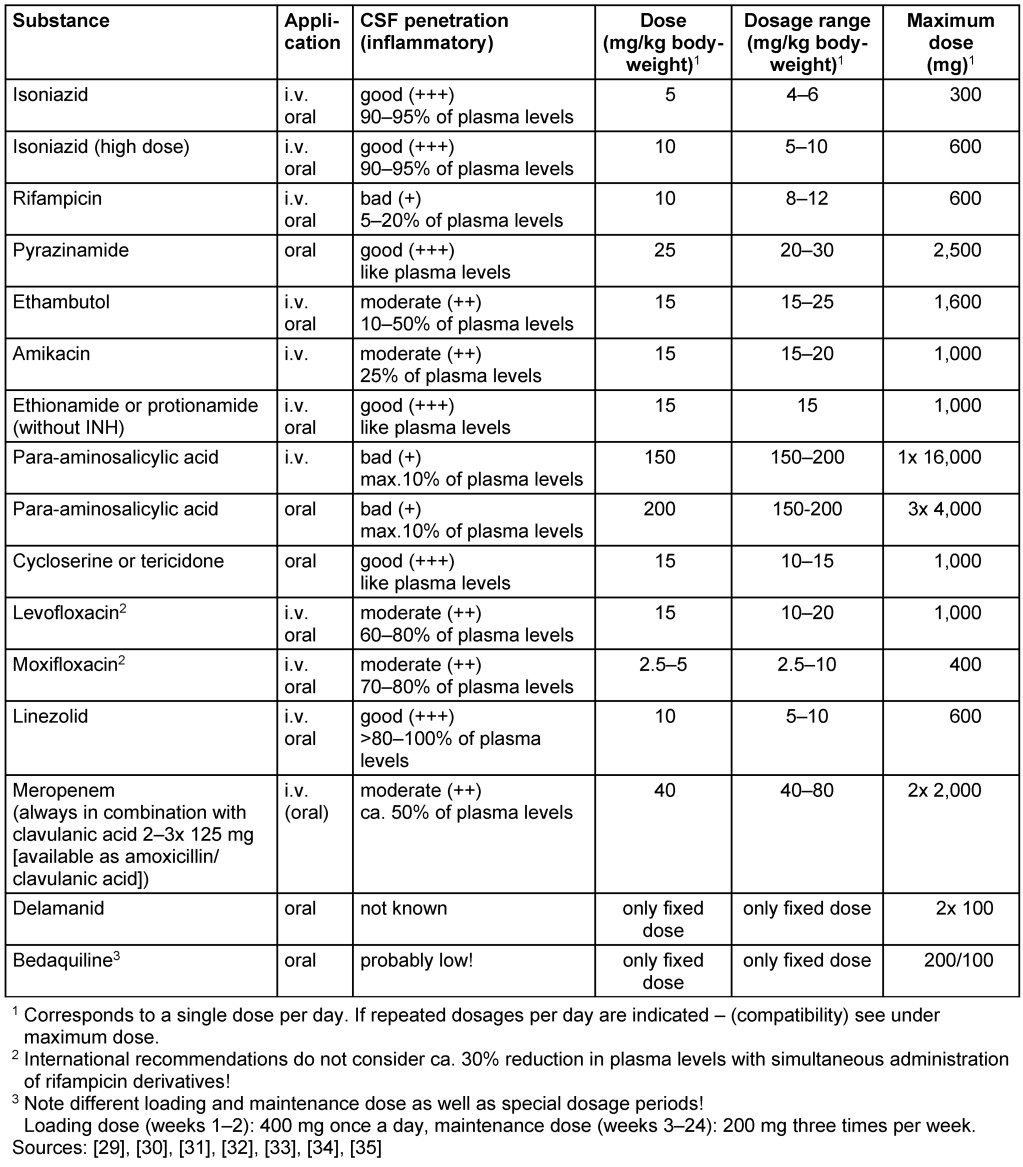
Tuberculous meningitis: Dosage recommendations according to national and international guidelines. Due to intolerance or resistance to standard substances of tuberculosis treatment in each case combination treatment with 4 effective tuberculosis drugs should be performed. If necessary, in addition to parenteral administration, only orally available agents (such as PZA, PAS, bedaquiline, delamanid) should be administered. These are therefore also listed in the table.
